# Co-treatment of Naringenin and Ketoprofen-RGD Suppresses Cell Proliferation via Calmodulin/PDE/cAMP/PKA Axis Pathway in Leukemia and Ovarian Cancer Cells

**DOI:** 10.5812/ijpr-136131

**Published:** 2023-06-19

**Authors:** Havva Afshari, Shokoofe Noori, Bahareh Shokri, Afshin Zarghi

**Affiliations:** 1 Department of Clinical Biochemistry, School of Medicine, Shahid Beheshti University of Medical Sciences, Tehran, Iran; 2 Department of Pharmaceutical Chemistry, School of Pharmacy, Shahid Beheshti University of Medical Sciences, Tehran, Iran

**Keywords:** Chronic Myelogenous Leukemia, Ovarian Cancer, Ketoprofen-RGD, Naringenin, Protein Kinase A

## Abstract

**Background:**

Naringenin (Nar) has anti-inflammatory and anticarcinogenic properties. Arginine-glycine- aspartate (RGD) is a tripeptidic sequence used as an integrin ligand and targeting system for delivering chemotherapeutic agents to cancer cells.

**Objectives:**

In this study, the inhibitory effects of Nar and ketoprofen-RGD on leukemia and ovarian cancer cells (K562 and SKOV3) were explored for the first time, focusing on their proliferation activity and their anti-inflammatory capacity.

**Methods:**

Analyses were conducted on the calmodulin (CaM)-dependent phosphodiesterase 1 (PDE1) activation by ketoprofen-RGD, Nar, and their combination. These drugs’ effects on protein kinase A (PKA) activation, intracellular cyclic adenosine monophosphate (cAMP) level, and PDE1 inhibition were identified. Later, it was also evaluated if ketoprofen-RGD alone or in combination with Nar had anti-inflammatory effects.

**Results:**

Nar improved the antagonizing consequences of ketoprofen-RGD on the CaM protein, which hinders PDE1, improving PKA activity and cAMP levels. A mixture of ketoprofen-RGD and Nar and ketoprofen-RGD alone diminished K562 and SKOV3 cell viability through the cAMP/PKA pathway by inhibiting PDE1 and CaM. These two compounds showed anti-inflammatory effects on both cell lines.

**Conclusions:**

This study indicated for the first time that combining ketoprofen-RGD and Nar can be a promising anti-inflammatory therapeutic regimen for treating leukemia and ovarian cancer.

## 1. Background

Ovarian cancer has the highest mortality rate compared to other gynecologic cancers. Chronic myeloid leukemia (CML) is a clonal myeloproliferative tumor characterized by the unregulated growth of myeloid cells (1, 2).

Calmodulin (CaM) is a Ca^2+^-binding molecule that has been highly conserved throughout biological evolution. It has been proven to have key functions in regulating apoptosis and promoting proliferation in many cancer cells (3, 4). Phosphodiesterases (PDEs) are pivotal enzymes important in moderating the cross-talk between cyclic adenosine monophosphate (cAMP) and Ca^2+^ signaling. PDE has several isoforms, of which PDE1 is a Ca^2+^/CaM-dependent enzyme. It has been reported that PDE1 inhibition could generate the induction of apoptosis in some cancer cells (5, 6). Furthermore, cAMP was discovered as one of the numerous obsolete signaling molecules to transform extracellular signals into characteristic cellular responses to activate protein kinase A (PKA), which functions in a variety of cellular events (7). Adenylyl cyclases synthesize cAMP from ATP, much as PDEs hydrolyze this second precursor to its inactive 5’-monophosphate. It has been previously demonstrated that the cAMP-dependent PKA is crucial for cell migration in several cells (8). Also, inflammation is a risk factor for cancer, and the inflammatory reaction is implicated in nearly all phases of tumor maturation. Inflammatory mediators, including tumor necrosis factor-alpha (TNF-α), interleukin-6 (IL-6), and interleukin-10 (IL-10), have been implicated in cancer metastasis (9).

Specific natural medicines, such as flavonoids or natural polyphenolic mixtures, have been shown to have broad pharmacological effects with low toxicity (10). Thus, several clinical trials have demonstrated that traditional medicine combined with chemotherapy or radiotherapy may help ovarian cancer patients (11). Flavonoids have long been recognized for their ability to halt the progression of carcinogenesis (12). Naringenin (Nar) is a flavonoid mostly extracted from citrus fruits. This compound has various useful pharmacological effects involving anticarcinogenic and anti-inflammatory effects (13, 14). Our previous in vitro studies demonstrated that Nar could impede cell proliferation and migration and generate cell cycle arrest and apoptosis in cancer cell lines (15).

Furthermore, as demonstrated in our previous study (16), the tripeptidic sequence arginine-glycine-aspartate (RGD) can be used as an integrin ligand and a targeting system for delivering chemotherapeutic agents to cancer cells. Ketoprofen is a nonsteroidal anti-inflammatory drug (NSAID) used to ease pain, including bone cancer pain (16). According to two early-stage clinical trials, ketoprofen is effective in treating lymphedema (17). However, there is limited information on its effectiveness in treating other types of cancer or its potential as a cancer treatment. Ketoprofen-RGD has shown promising anticancer properties, particularly in breast cancer stem-like cells (BCSCs) and their parental cells (18).

## 2. Objectives

This study aimed to investigate the effects of Nar on ketoprofen-RGD anticancer activity via the CaM/PDE/cAMP/PKA signaling pathway, as well as biomarkers of inflammation, and to estimate the value of Nar and ketoprofen-RGD in K562 and SKOV3 to investigate novel strategies for ovarian cancer and CML treatment.

## 3. Methods

### 3.1. Ketoprofen-RGD Preparation

Dr. Afshin Zarghi from the School of Pharmacy, Shahid Beheshti University of Medical Sciences, Tehran, Iran, generously provided ketoprofen-RGD.

#### 3.1.1. Ketoprofen-RGD Synthesis

In summary, synthesizing the ketoprofen-RGD conjugate involves placing 0.5 g of Rink amide resin in a reactor, suspending it in dimethylformamide (DMF) under nitrogen, adding RGD to the reactor, and stirring for 2 hours, then adding ketoprofen to the reactor and stirring for 12 hours, filtering the resin and washing it with DMF, dichloromethane (DCM), and methanol deprotect the N-terminal amine of the peptide with trifluoroacetic acid (TFA). Purifying the product was done using high-performance liquid chromatography (HPLC). The product was analyzed through mass spectrometry and nuclear magnetic resonance (NMR) spectroscopy (19). The title compound was obtained as a white solid with a yield of 65%.

**Figure 1.**
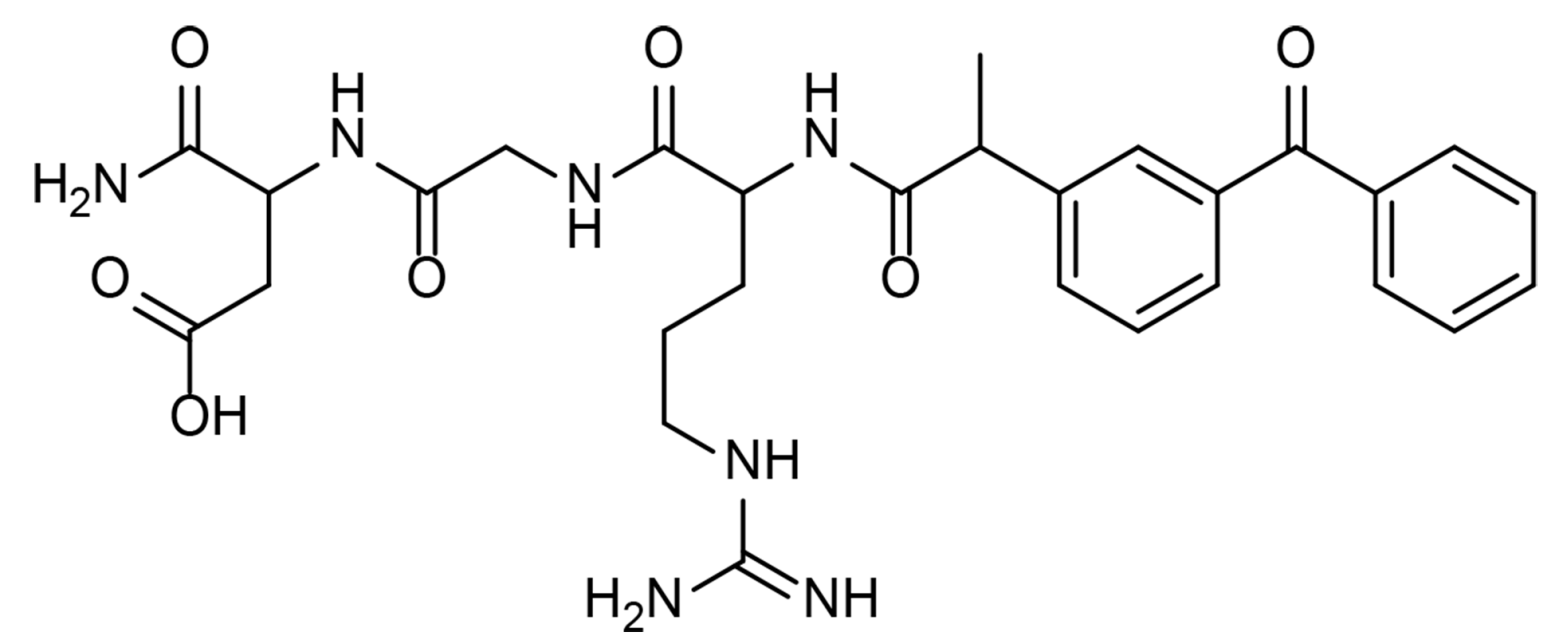
The structure of ketoprofen conjugated to arginine-glycine-aspartic acid (RGD)

^1^H NMR (500 MHz, DMSO-*d*_*6*_) δ: 1.32 - 1.33(d, *J* = 0.01, ^3^H, CH_3_), 1.45 - 1.46 (m, ^2^H, CH_2_), 1.64 - 1.82 (m, ^2^H, CH_2_), 2.5 (s, ^1^H, NH), 2.96,3.07 (m, ^2^H, CH_2_), 3.64 - 3.69 (m, ^2^H, CH_2_), 3.82 - 3.85 (m, ^1^H, CH), 4.15 - 4.16 (m, ^2^H, CH_2_), 4.26 - 4.27 (m, ^1^H, CH), 4.38 - 4.43 (m, ^1^H, CH), 7.05 - 8.31(^15^H, NH, NH_2_ and aromatic hydrogen), 11.8 (br s, ^1^H, OH) ppm; IR (KBr): υ (cm^-1^) 1656, 3330; LC-MS (ESI) m/z (M + H)^+^ calcd for C_28_H_35_N_7_O_7_: 581, found: 582.2 (Figure 1).

### 3.2. Molecular Modeling Studies

Docking studies were performed using AutoDock software version 4.0 to search for favorable binding configurations between the small flexible ligands (Nar and ketoprofen-RGD) and the rigid protein to predict their interactions. These studies were performed based on the high-resolution crystal structure of the CaM receptor (Protein Data Bank (PDB) code: 1QIW) retrieved from the RCSB Protein Data Bank. The co-crystallized ligand (DPD) and water molecules were removed from the protein. Kollman charges were added, nonpolar hydrogens were merged, and AutoDock 4 atom type was assigned to achieve the PDBQT format of the protein. The ligand structures were minimized using HyperChem8.0 (MM+ method) and then converted to PDBQT file format with AutoDock tools. A docking grid box was built with (30 × 30 × 30) surrounding the co-crystallized ligand. The Lamarckian genetic search algorithm was employed, and the docking run was set to 100. Protein residues with distances greater than 6.0 Å from the docking box were removed for efficiency. The quality of the docked structures was evaluated by measuring the intermolecular energy of the ligand-enzyme assembly.

### 3.3. Determination of Kinetic Parameters

ΔG (H_2_O) was used as a suitable parameter to estimate CaM protein stability in response to ketoprofen-RGD and Nar. The following formula was used to calculate the diversity between ΔG of native and denatured conformations of CaM protein (20):


$$\Delta G\;=\;-RT\ln\frac{F_{d}}{1-F_{d}}$$

$$F_{d}=\frac{Y_{n}-Y_{obs}}{Y_{n}-Y_{d}}$$

$$\ln\frac{Y_{n}-Y_{obs}}{Y_{obs}-Y_{d}}=\;-RT\ln K$$

In this equation, R stands for the gas constant, T for the actual temperature, Y_obs_ for the CaM fluorescence emission intensity at various ketoprofen-RGD and Nar concentrations, and Y_n_ and Y_d_ for the values of native and denatured conditions, respectively. The following equation demonstrated a linear relationship between the ΔG alterations and the concentrations of ketoprofen-RGD and Nar (20):


$$\Delta G\;=\;\Delta G\;(H_{2}O)\;-\;m\;[D]$$

In this formula, ΔG (H_2_O) represents ΔG at zero ligand concentration, m is ongoing for the reliance of ΔG on ligand concentration, and [D] is the ligand concentration.

Lineweaver-Burk plots were developed due to a kinetic analysis of CaM-dependent PDE1 restraint in response to ketoprofen-RGD and Nar. Using the previously described plots, Km values were determined in both the presence and absence of both ligands. According to these plots, the point at which the lines of the Lineweaver-Burk plot cross the y-axis corresponds to the maximum velocity (V_max_) of PDE1 activity in the presence or absence of the two drugs.

### 3.4. Cell Culture

In the present investigation, SKOV3 and K562 were used to investigate the anti-proliferative influences of our tested compounds. These cell lines were bought from the Pasteur Institute (Tehran, Iran). First, the cells were grown in Roswell Park Memorial Institute (RPMI) 1640 culture medium (Thermo Fisher Scientific in the United States), which was supplemented with 10% FBS, 100 mg/mL of streptomycin, and 100 units/mL of penicillin. The cell lines were grown in an incubator at 37°C and 5% CO_2_.

### 3.5. MTT Assay

The MTT assay was employed to assess the K562 and SKOV3 cells’ viability following ketoprofen-RGD and Nar treatments. Accordingly, the cells were harvested and seeded in 96-well plates (6000 cells per well). After 24 h, the cells were treated with different concentrations of ketoprofen-RGD (0 - 5 μM) and Nar (50 μM) for 48 h. Then, a 10% MTT solution was added to each well and incubated for 4 h. Later, 100 μL of dimethyl sulfoxide (DMSO) (Sigma-Aldrich Company, USA) was added to dissolve the formazan crystals, and the absorbance was recorded at 570 nm by an ELISA reader.

### 3.6. Measurement of cAMP Level

An enzyme immunoassay kit (Stratagene, La Jolla, CA) determined the total concentration of cAMP molecules in K562 and SKOV3 cells. In summary, the cells in the treatment group were given the concentrations mentioned above of ketoprofen-RGD and Nar, whereas the cells in the control group were given a culture medium. The total cAMP level in K562 and SKOV3 cells was then calculated using the kit’s protocols.

### 3.7. Measurement of PKA Activity

The PKA enzyme’s activity was measured using a colorimetric assay kit (BioVision, USA). The treated K562 and SKOV3 cells were resuspended in lysis buffer (50 mM Tris-HCl, 2.5 mM EDTA, 1 mM MgCl_2_, 10 mM NaF, 10% glycerol, pH = 7.2) after being rinsed twice with phosphate-buffered saline (PBS) buffer (pH =7.4). The manufacturer’s procedures were then used to determine PKA’s activity.

### 3.8. PKA Inhibition Effects on the K562 and SKOV3 Cells’ Proliferation

To evaluate the impact of PKA inhibition on the proliferation of K562 and SKOV3 cells, a PKA inhibitor, Rp-cAMP (Biolog Life Science Institute, Bremen, Germany), was used. Accordingly, the cells were exposed to Rp-cAMP (100 μM) for 20 min and then incubated with ketoprofen-RGD (0 - 5 μM) alone or combined with Nar (50 μM).

### 3.9. Anti-inflammatory Effects of Ketoprofen-RGD and Nar on k562 and SKOV3 Cells

To explore the anti-inflammatory impacts of ketoprofen-RGD and Nar, k562 and SKOV3 cells were pretreated with lipopolysaccharide (LPS) (10 g/mL) for 4 h, an inducer of inflammatory responses. Then, the cells were treated with ketoprofen-RGD (3 μM) alone and in combination with Nar (50 μM). The cells were exposed to forskolin (FSK, 0.5 μM) as a standard anti-inflammatory agent (18). Subsequently, the ELISA method was used to measure the levels of some pro-inflammatory cytokines.

### 3.10. Western Blot Analysis

K562 and SKOV3 cells were seeded into six-well plates and incubated with different concentrations of compounds. Then, cells were lysed in RIPA buffer (Thomas Scientific Inc., USA) following two PBS washes. The centrifuged lysates’ supernatant was utilized for Western blot analysis. After loading into a 10% SDS-PAGE with 40 g of total protein per lane, the samples were electroblotted onto a polyvinylidene fluoride (PVDF) membrane. Membrane blocking was accomplished with 5% non-fat milk for an hour at room temperature. The membranes were then incubated with the antibodies against IκBα and β-actin (Sigma-Aldrich, Germany) at 4°C for one more night. The membranes were covered with a secondary antibody (Santa Cruz Biotechnology, UK) conjugated to horseradish peroxidase (HRP) and remained in the dark at room temperature for an hour. A chemiluminescent kit was employed to detect immunoblots, and the thickness of the visible bands was measured using ImageJ software.

### 3.11. Statistical Analysis

K-S and Levene’s statistical tests were used in the same order to assess the normality of variances. A one-way analysis of variance (ANOVA) or Mann-Whitney nonparametric test was applied to the study’s findings. The threshold for statistical significance between the groups was set at P < 0.05. Using SPSS software version 22, all data were examined.

## 4. Results

### 4.1. Molecular Modeling Studies

The CaM receptor pocket is formed by two subunits (A and B) occupied by the co-crystallized inhibitor (DPD). The docking studies were performed on subunit B of calmodulin.

**Figure 2.**
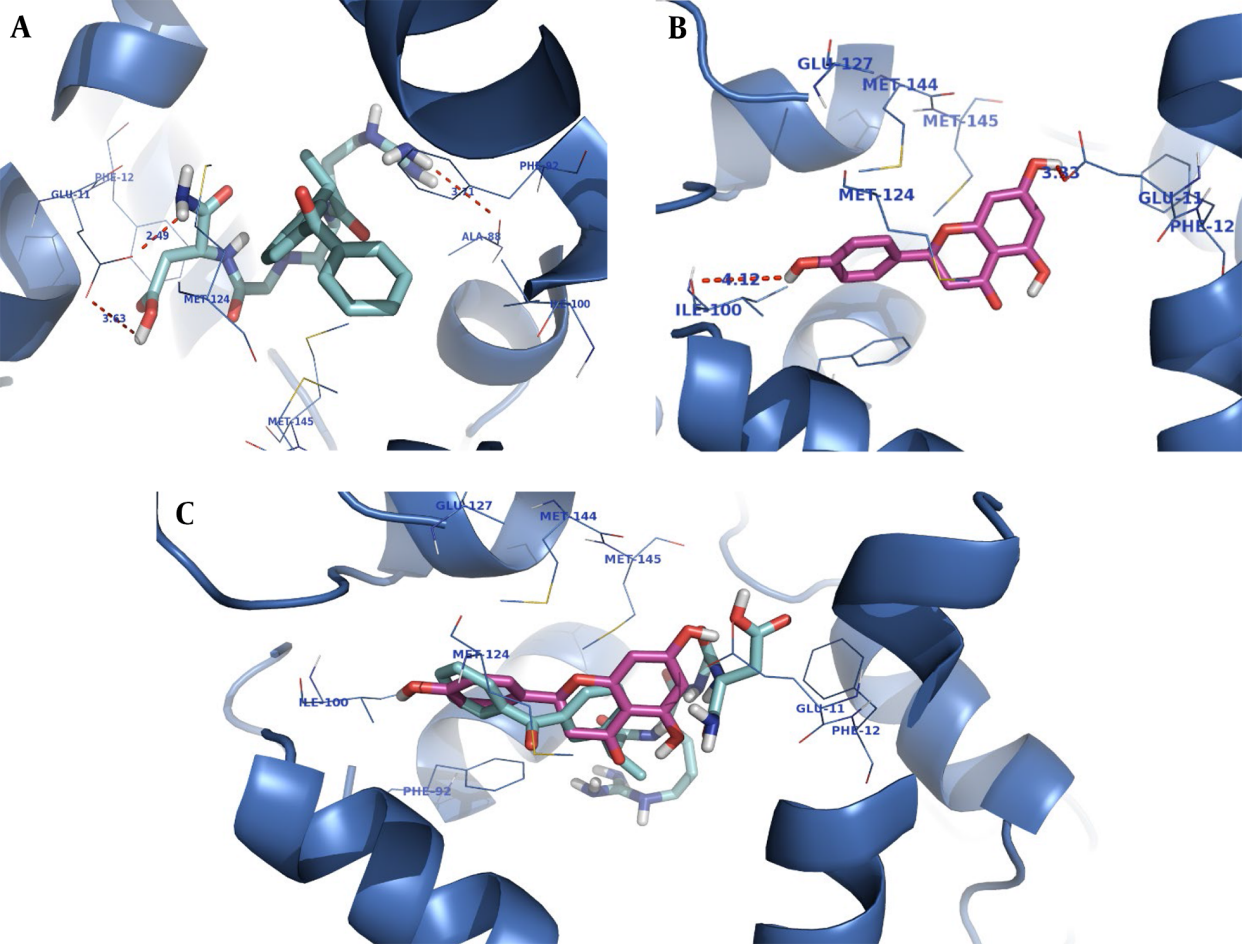
Docking of ketoprofen-arginine-glycine-aspartate (RGD) (A), naringenin (Nar) (B), and superimposition of F ketoprofen-RGD on Nar (C) in the calmodulin (CaM) active site

The orientation of the compounds ketoprofen-RGD and naringenin in the CaM active site is shown in Figure 2. Both compounds occupy the same space, and their structures nicely fit into the same hydrophobic pocket of the CaM active site, containing Phe92, Met124, Met144, and Met145 as is occupied by the co-crystallized ligand. As demonstrated in Figure 2, the guanidine residue of ketoprofen-RGD plays a role as a hydrogen bond donor and forms a hydrogen bond with the oxygen atom of Ala88. The Glu11 residue of the active site has come into contact with the aspartic acid residue of ketoprofen-RGD. Therefore, the carboxylic acids of both Glu11 and ketoprofen-RGD have hydrogen bond interactions. Furthermore, the amide group of ketoprofen-RGD can interact with Glu11 through hydrogen bonding.

The naringenin OH groups form hydrogen bonds with the Glu11 side chain carboxylic acid and the Ile100 oxygen atom. Also, the naringenin and ketoprofen-RGD could participate in hydrophobic interactions and π-π interactions with hydrophobic residues and aromatic rings of the active site.

### 4.2. Effect of Ketoprofen-RGD and Nar on Calmodulin

**Figure 3.**
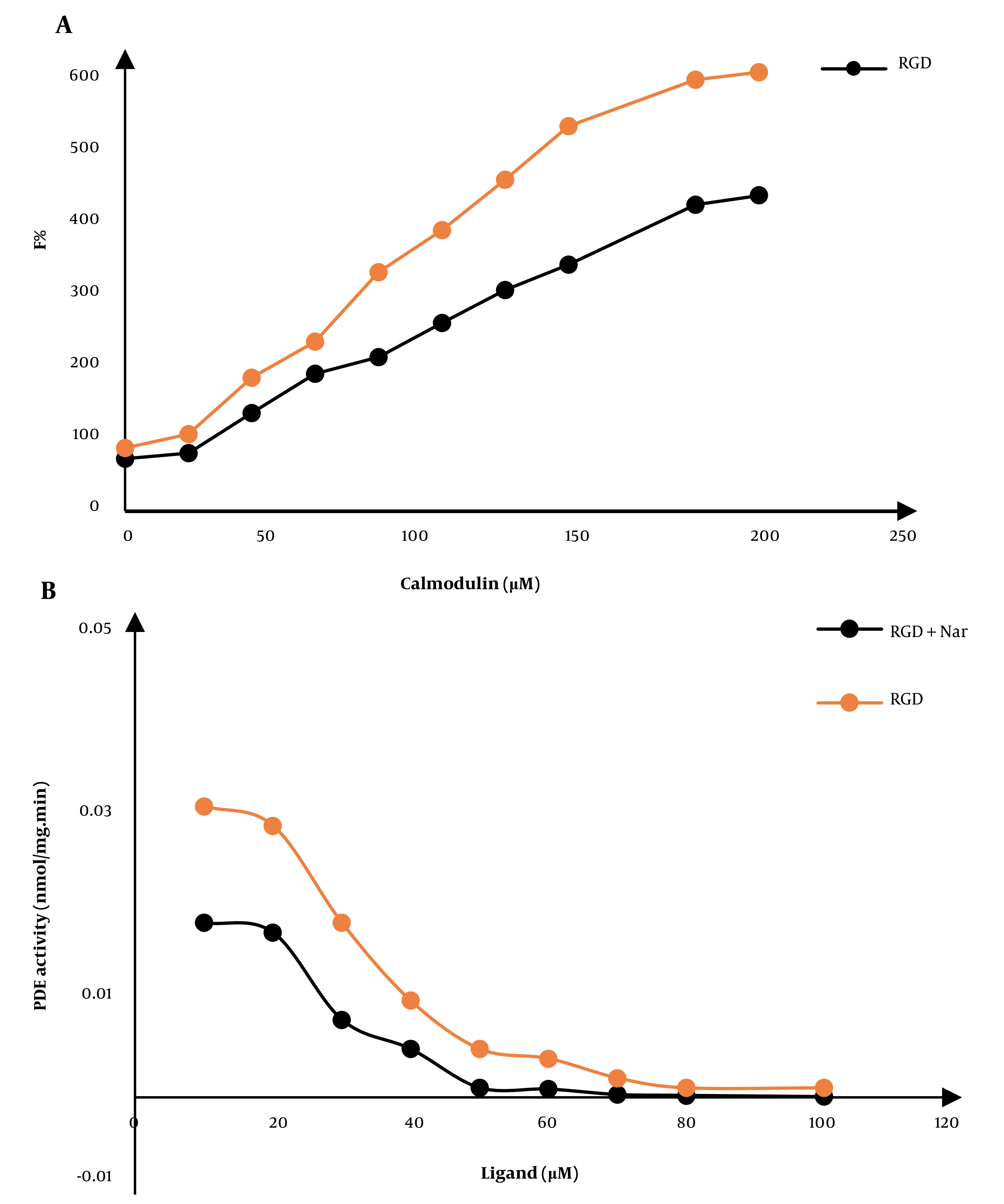
A, The fluorescence spectra of calmodulin (CaM) at 0.043 mg/mL concentration in a 40 mM Tris-HCl buffer, pH 7.4 at 25°C. The gradual increase in the fluorescence of CaM in the free form and the presence of 10 μM of ketoprofen- arginine-glycine-aspartate (RGD), naringenin (Nar), and their combination are shown. B, The CaM-dependent activity of phosphodiesterase 1 (PDE1) in response to various doses of ketoprofen-RGD, Nar, and their combination

The changes in the fluorescence emission spectra of CaM could show an interaction between the two compounds, ketoprofen-RGD and Nar, with CaM. We also observed a ligand-induced conformational change in CaM that represents the inhibitory influences of these two compounds on this protein. The maximum fluorescence emissions of CaM were also increased due to combining the two compounds. For example, the combination of ketoprofen-RGD and Nar had a higher effect on the maximum emission of CaM than did ketoprofen-RGD and Nar alone (Figure 3A).

### 4.3. Effect of Ketoprofen-RGD and Nar on Calmodulin Stability

**Figure 4.**
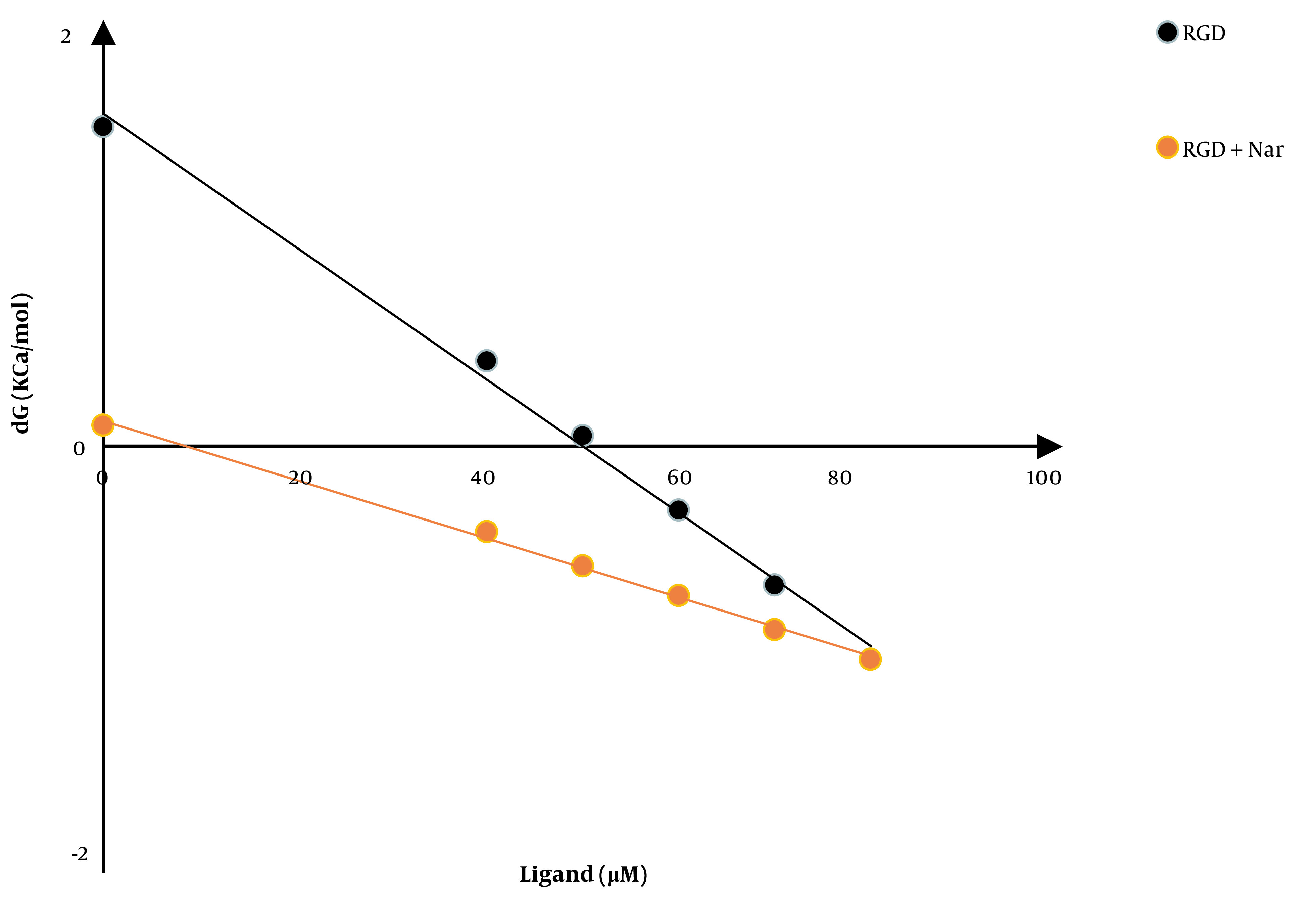
The differences in the ΔG of calmodulin versus different doses of ketoprofen- arginine-glycine-aspartate (RGD), naringenin (Nar), and their mixture

Another experiment evaluated the CaM stability in response to ketoprofen-RGD, Nar, and their combination. Figure 4 shows the CaM (ΔG) alterations following the binding of ketoprofen-RGD and Nar alone and their combination with the CaM protein. According to the linear plots, the computed ΔG (H_2_O) was 1.5, 2.4, and 0.1 kcal mol^-1^ in the presence of ketoprofen-RGD, Nar, and their combination, compared to 7.1 kcal mol^-1^ in the absence of these two substances (Table 1).

**Table 1.** Calculated Parameters of Calmodulin Stability Against Naringenin, Ketoprofen-Arginine-Glycine-Aspartate, and Naringenin + Ketoprofen- Arginine-Glycine-Aspartate

Ligand	Concentration (μM)	∆G (H_2_O) (kCal/mol)	M (kCal/mol/μM)	D_1/2_ (μM)	R^2^
**Calmodulin**	5	7.1	0.101	69.7	0.99
**Nar**	50	2.4	0.042	55.3	0.99
**Ketoprofen-RGD**	5	1.5	0.0313	49.84	0.99
**Nar + Ketoprofen-RGD**	50+5	0.1	0.0138	8.69	0.99

Abbreviations: Nar, naringenin; RGD, arginine-glycine-aspartate

### 4.4. Effect of Ketoprofen-RGD and Nar on the PDE-1 Activity

In the presence of growing concentrations of ketoprofen-RGD, Nar, and their combinations, the function of the PDE-1 enzyme was quantified. Hydrolyzing cAMP to AMP with a spectrophotometer is considered the best way to measure PDE-1 activity. Ultimately, the activity of PDE-1 was calculated and plotted against different concentrations of ketamine-RGD and Nar. (Figure 3B).

### 4.5. Analysis of the PDE1 Activity’s Kinetics in Response to Ketoprofen-RGD and Nar

**Figure 5.**
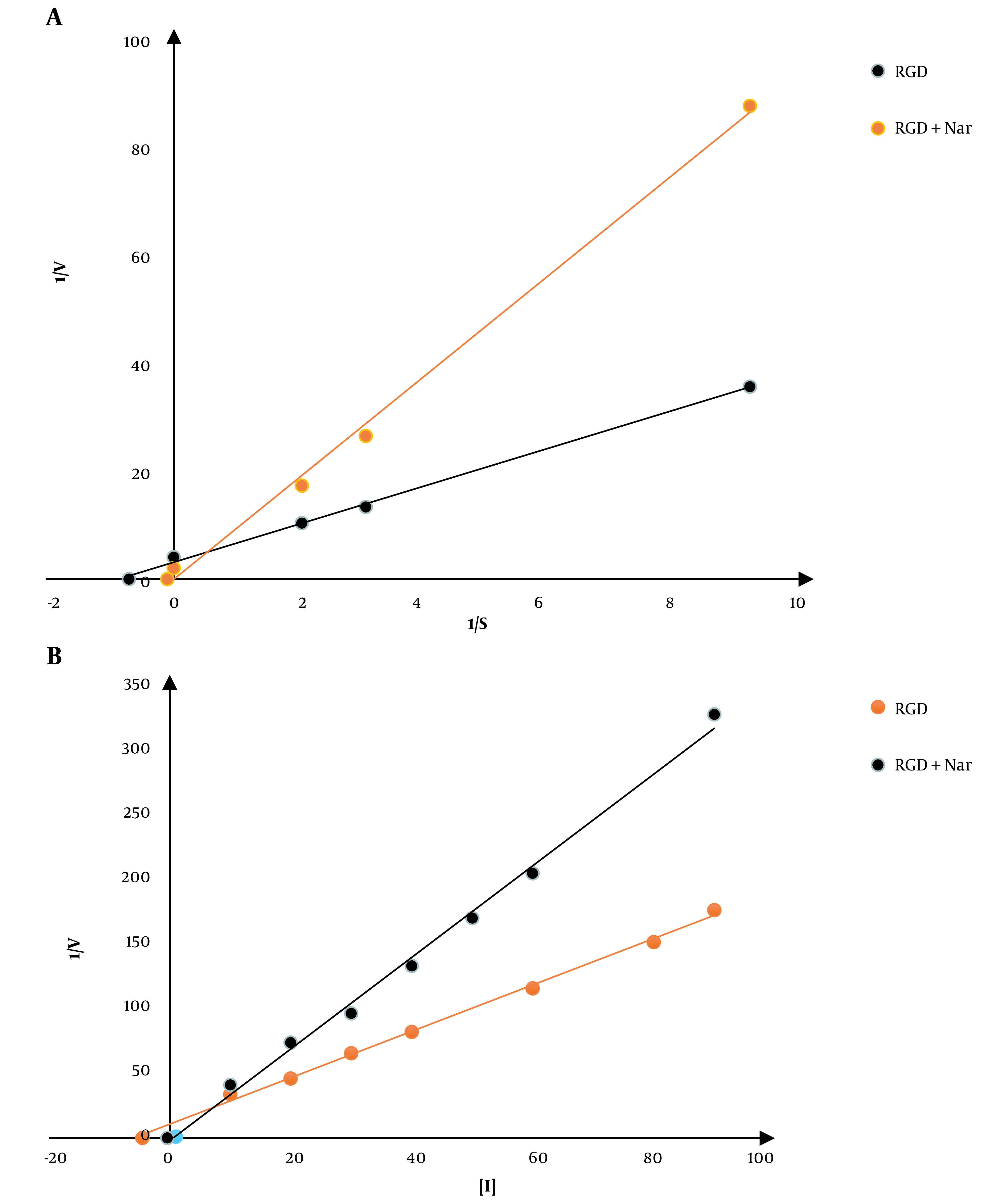
A, A Lineweaver-Burk plot is used to examine phosphodiesterase 1 (PDE1) conditioning and the mechanism by which the enzyme is restrained using ketoprofen-arginine-glycine-aspartate (RGD) and naringenin (Nar). The restriction was achieved using 5 μM ketoprofen-RGD, 50 μM Nar, and their combination at various cyclic adenosine monophosphate (cAMP) concentrations (the calmodulin (CaM) concentration in all reactions remained constant at 100 μM). B, Dixon plot: Several concentrations of the two drugs and their combination were used to achieve inhibition.

First of all, the kinetic constant of PDE1 was calculated. The Km value of PDE1 in the absence of both ligands was 0.05 ± 0.001 μM; however, by the addition of ketoprofen-RGD and Nar, either alone or in their combination, the Km values were increased to 0.05, 0.1, and 10, μM respectively. These results showed that ketoprofen-RGD and Nar could increase the Km values in a concentration-dependent manner. No significant changes in Vmax values accompanied this increase in Km values. The following is the effectiveness of the combination of ketoprofen-RGD and Nar in increasing Km values: Ketoprofen-RGD + Nar > ketoprofen-RGD > Nar. In line with the Lineweaver-Burk plots, the two ligands, either alone or in association, competitively inhibited PDE1. The Ki constant was used to evaluate the effectiveness of the two inhibitors. This variable demonstrates the relationship of the inhibitor to a distinct enzyme. The calculated Ki values for ketoprofen-RGD, Nar alone, and their combination were 4.5, 10, and 0.4, respectively (Figure 5).

### 4.6. Ketoprofen-RGD and Nar Effects on K562 and SKOV3 Cell Proliferation

**Figure 6.**
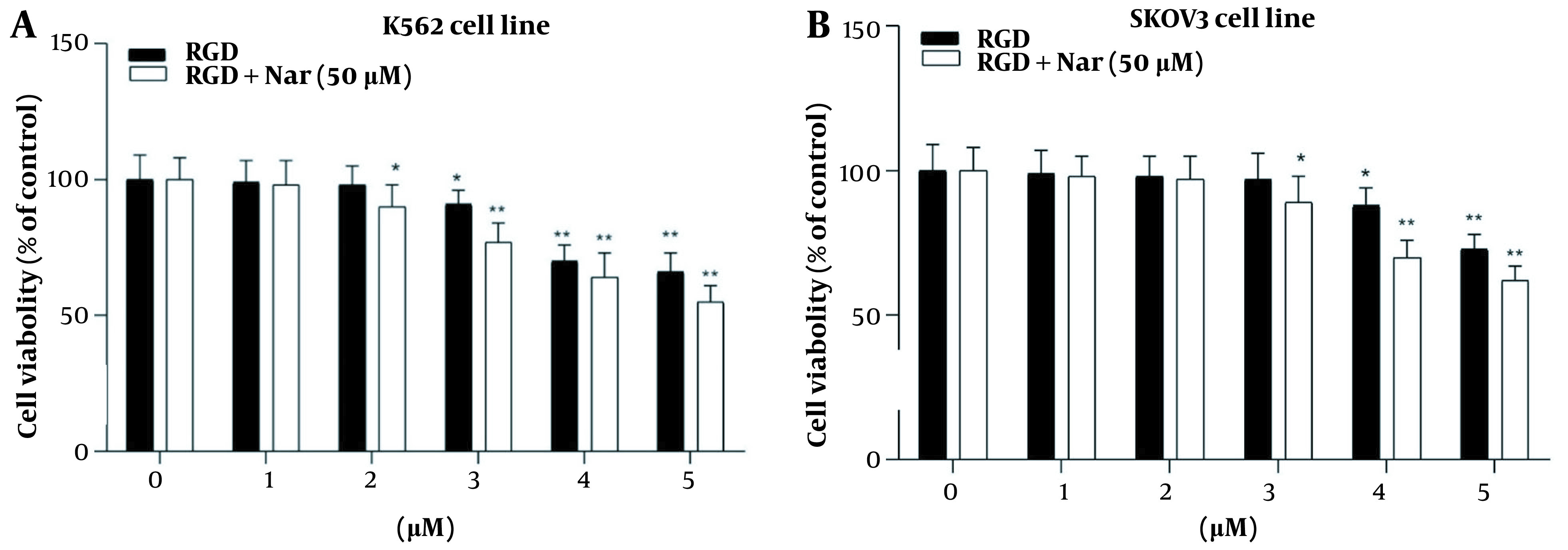
The effects of ketoprofen-arginine-glycine-aspartate (RGD) and its combination with Nar on the viability of the K562 (A), and SKOV3 (B) cell lines (* P < 0.05, ** P < 0.01 vs. control)

The treated K562 and SKOV3 cells with different concentrations of ketoprofen-RGD (0 - 5 μM) alone or in combination with Nar (50 μM) indicated that ketoprofen-RGD significantly declined the viability of these tumor cells at concentrations greater than 2 μM. Furthermore, combining ketoprofen-RGD with Nar had a more significant effect (P < 0.01) on reducing K562 and SKOV3 cells’ proliferation compared with untreated controls. This may suggest that the combination therapeutic approach was more useful than the single agent in inhibiting the viability of K562 and SKOV3 cells (Figure 6).

### 4.7. Effects of ketoprofen-RGD and Nar on PKA Activity and Intracellular Levels of cAMP

**Figure 7.**
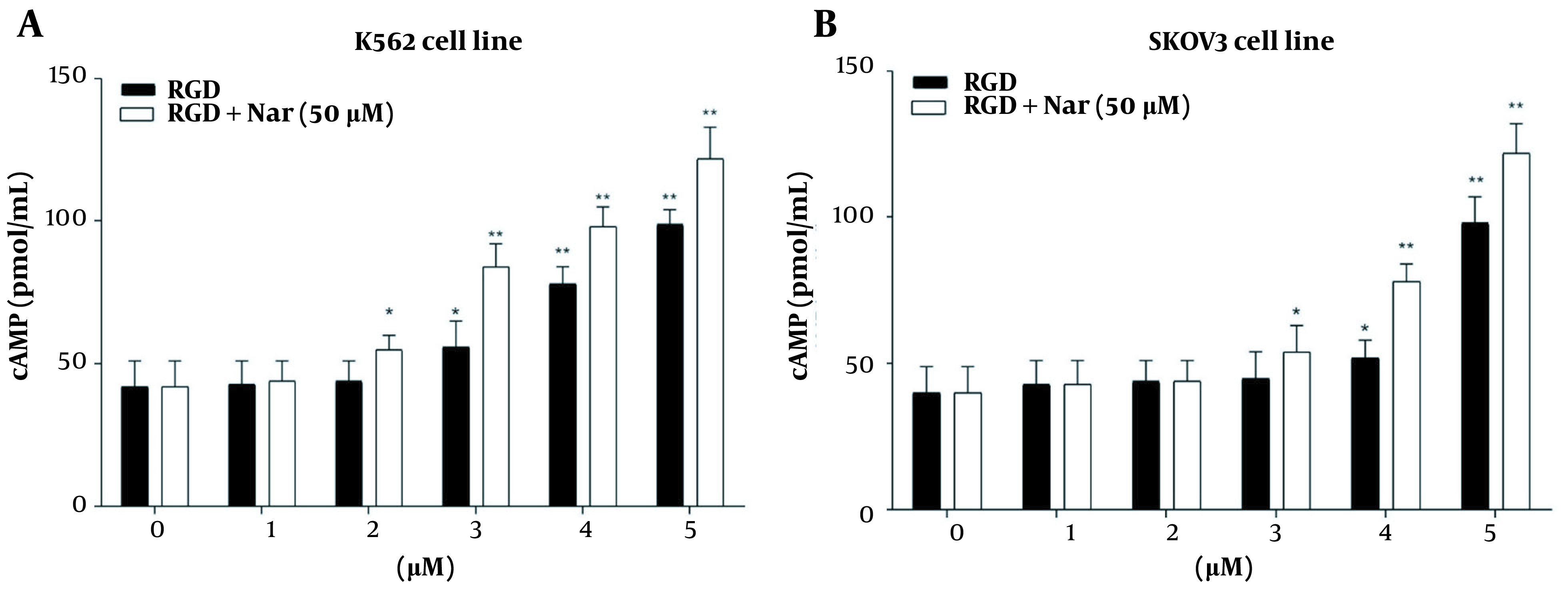
The effects of treating K562 (A), and SKOV3 (B) cell lines with various concentrations of ketoprofen-arginine-glycine-aspartate (RGD) and its combination with naringenin (Nar) on the cyclic adenosine monophosphate (cAMP) level. * P < 0.05 and ** P < 0.01 compared with the control group

The potential effects of ketoprofen-RGD (0 - 5 μM) alone and combined with Nar (50 μM) on PKA activity and cAMP level in K562 and SKOV3 cells were examined using the enzyme immunoassay. According to the obtained results, intracellular cAMP accumulation was observed in K562 (P < 0.01) and SKOV3 (P < 0.01) cells in response to ketoprofen-RGD alone and its combination with Nar. This accumulation was considerable at doses of more than 2 μM (Figure 7).

**Figure 8.**
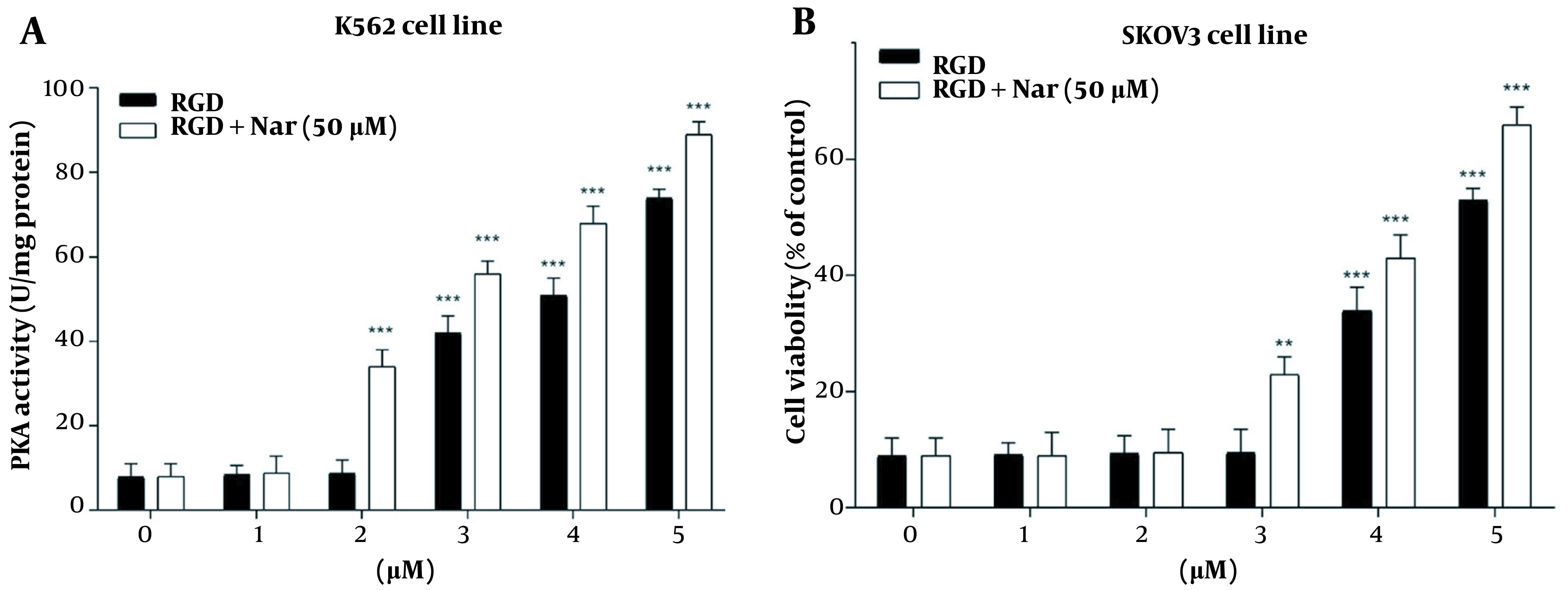
The activity of protein kinase A (PKA) in K562 (A), and SKOV3 (B) cells in response to ketoprofen-arginine-glycine-aspartate (RGD) and its combination with naringenin (Nar). * P < 0.05, ** P < 0.01, and *** P < 0.001 compared with the control group

Moreover, treating K562 and SKOV3 cells with either ketoprofen-RGD or its combination with Nar revealed a remarkable induction in the activity of PKA in comparison to the control group. Of note, the combination of the two compounds had a non-significantly higher effect on the cAMP levels and activity of PKA (Figure 8).

### 4.8. Effect of Ketoprofen-RGD and Nar on Cell Growth Inhibition via Activation of PKA by cAMP

**Figure 9.**
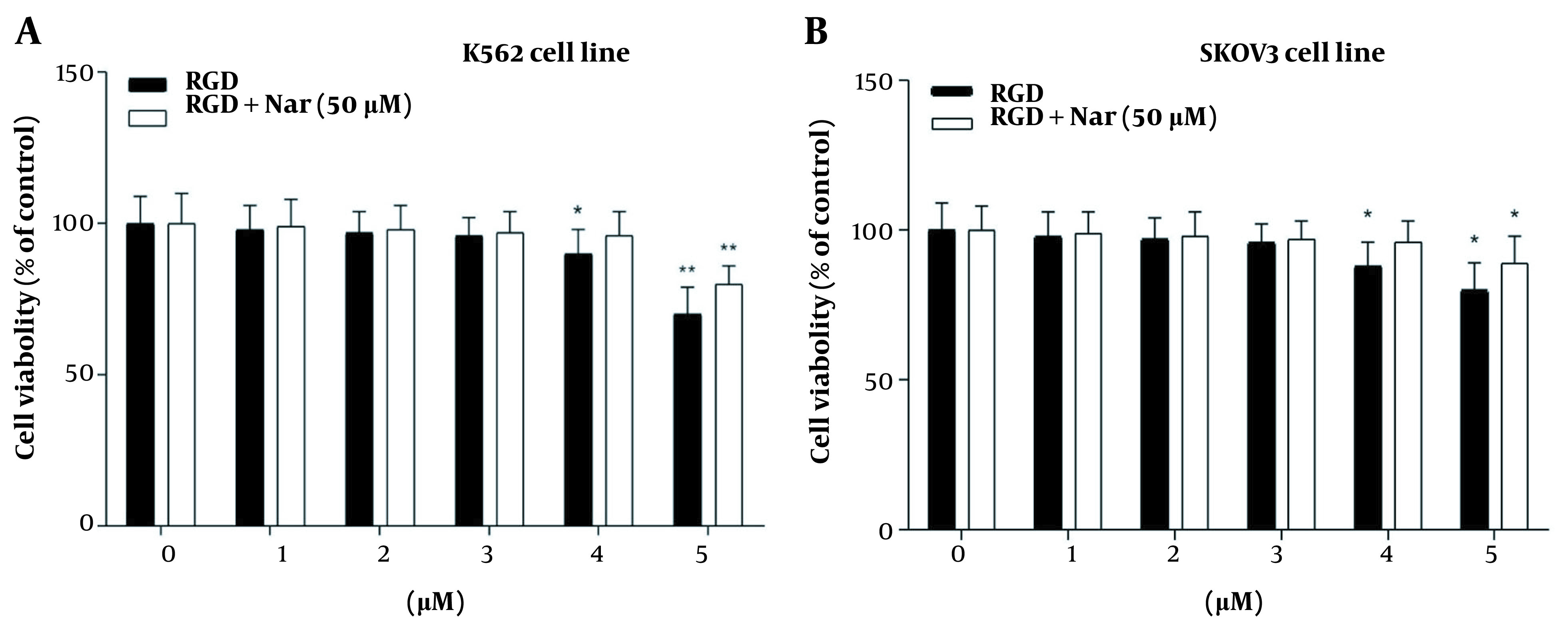
The effects of pretreating K562 (A), and SKOV3 (B) cells with Rp-cAMP, a common protein kinase A (PKA) inhibitor, on cell viability when ketoprofen-arginine-glycine-aspartate (RGD) is present alone or in combination with Nar. Data are presented as the mean ± SEM of three independent experiments. * P < 0.05, ** P < 0.01 vs. control group

In the final step, the K562 and SKOV3 cells were pretreated with an exact inhibitor of PKA (Rp-cAMP; 10 μM) and thus treated with ketoprofen-RGD alone (0 - 5 μM), and its combination with Nar (50 μM). This experiment was conducted to unravel the mechanism of K562 and SKOV3 cell growth inhibition. Figure 9 represents that pretreatment of SKOV3 and K562 cells with Rp-cAMP and treatment of them with the two compounds particularly hampered the proliferation of K562 (P < 0.05) and SKOV3 cells (P < 0.01) as compared with controls. This result suggests that the inhibition of K562 and SKOV3 cells’ proliferation by ketoprofen-RGD and Nar was moderated by PDE inhibition and the consequential activation of PKA due to the elevation of cAMP within these cancer cells.

### 4.9. Effects of Ketoprofen-RGD and Nar on the Level of the Pro-inflammatory Cytokine

**Figure 10.**
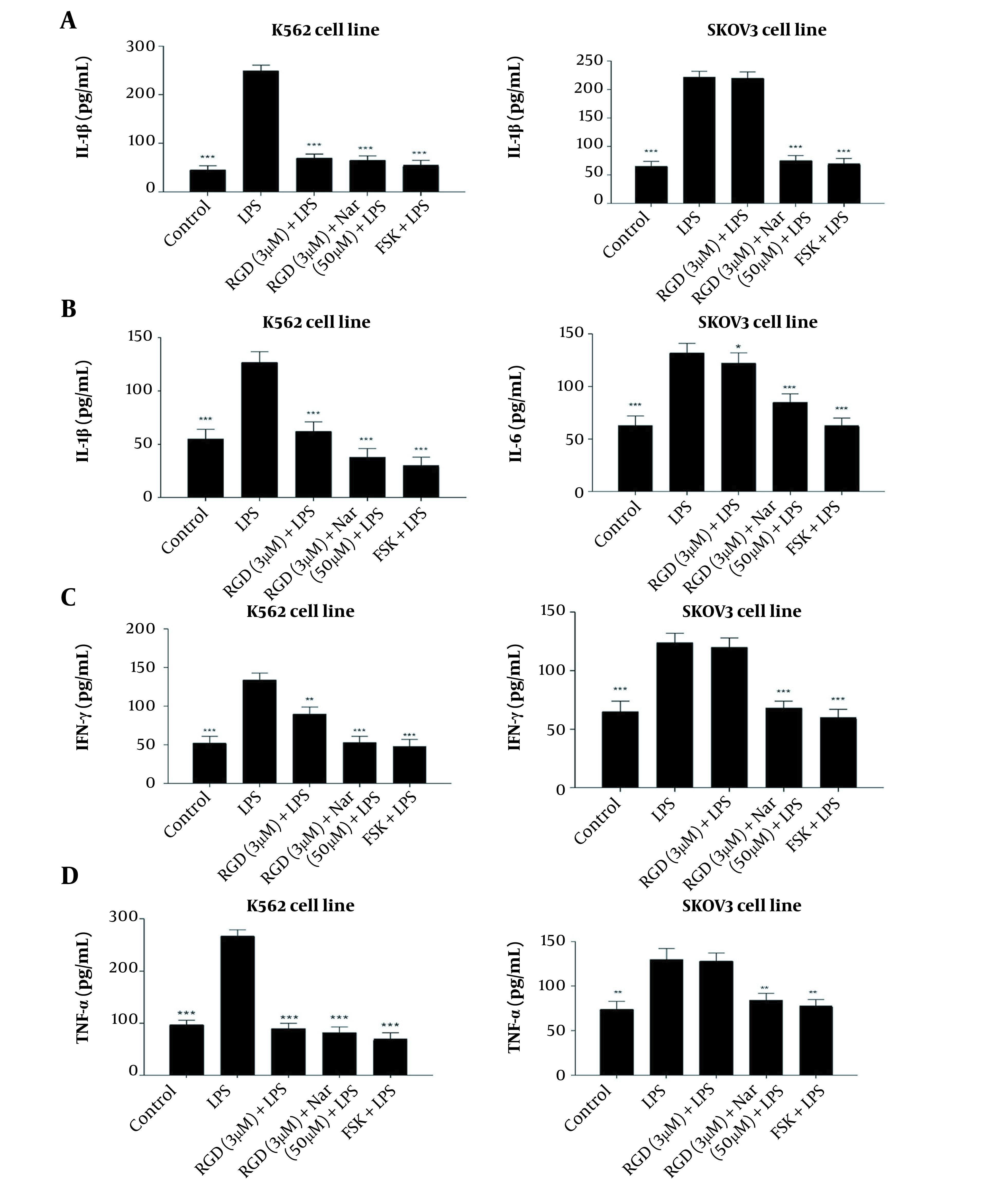
The effects of ketoprofen-arginine-glycine-aspartate (RGD) alone (3 μM) and its combination with Nar (50 μM) on the levels of interleukin-1β (IL-1β) (A), interleukin-6 (IL-6) (B), IFNγ (C), and tumor necrosis factor-alpha (TNF-α) (D) in K562 and SKOV3 cells. All data are expressed as mean ± SEM of three independent experiments. * P < 0.05, ** P < 0.01, *** P < 0.001 vs. lipopolysaccharide (LPS) group

As shown in Figure 10A - D, treating the K562 and SKOV3 cell lines with LPS as an inflammation inducer significantly enhanced the concentrations of IL-6, IL-1β, IFNγ, and TNF-α compared to untreated control cells. However, the levels of all the above-mentioned cytokines were significantly decreased in response to the treatment of K562 and SKOV3 cells with ketoprofen-RGD (3 μM) alone (P < 0.05) and its combination with Nar (50 μM) (P < 0.01). Interestingly, the decreases in the levels of these cytokines in reaction to these two drugs were comparable with the results obtained from the FSK treatment.

### 4.10. Effect of Ketoprofen-RGD and Nar on IκBα Protein Expression

**Figure 11.**
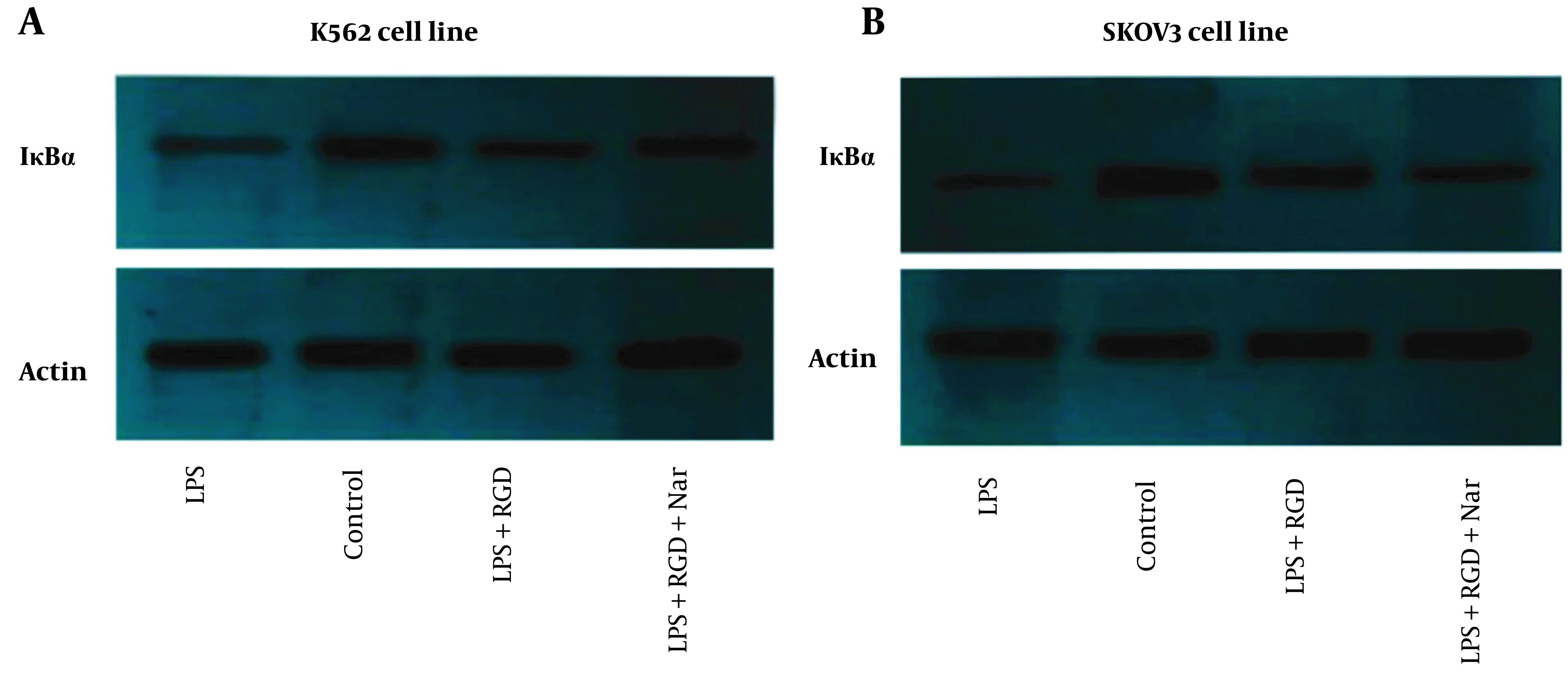
The effects of ketoprofen- arginine-glycine-aspartate (RGD) and naringenin (Nar) on IκBα protein expression in K562 (A), and SKOV3 (B) cell lines

As demonstrated in Figure 11A and B, the effects of Nar alone or combined with ketoprofen-RGD on IκBα protein expression in LPS-induced cells were examined. The western blotting results indicated that LPS caused a remarkable reduction in IκBα expression compared to the control in K562 and SKOV3 cell lines. In contrast, ketoprofen-RGD boosted the IκBα expression and reduced IκBα degradation by LPS in both cell lines. As Figure 11 shows, cells co-treated with Nar and ketoprofen-RGD further increased the IκBα protein expression in both cell lines compared to treatment with ketoprofen-RGD alone. β-actin was used as an internal control.

## 5. Discussion

CaM plays several key roles in the regulation of different characteristics of cancer cells, such as proliferation and apoptosis (21). PDE1 modulates the apoptosis and proliferation of cancer cells by inhibiting CaM-dependent phosphodiesterase (22, 23). Thus, inhibition of CaM by its antagonists has been revealed to impede the progression of cancer (24, 25). PDE1 inhibitors have been unraveled as targeted anticancer agents that can suppress the growth of cancer cells with no harmful effect on normal cells (26). Our previous investigation showed that ketoprofen-RGD could be considered a promising anticancer agent with pro-apoptotic properties against breast cancer cells (18).

Nar, as a polyphenolic flavonoid, has been indicated to have anticancer effects by inducing apoptosis and suppressing proliferation in breast cancer cells (27, 28). The present results indicated for the first time that the combination of ketoprofen-RGD and Nar caused strong conformational changes in the CaM protein. This effect was significantly higher than that of ketoprofen-RGD or Nar alone. This aligns with findings by Shi et al., which revealed that Nar could improve curcumin-induced apoptosis by modifying the activation of Akt, ERK, JNK, and p53 pathways in THP-1 cells (29). This study showed that the combination of ketoprofen-RGD and Nar was more efficacious than ketoprofen-RGD and Nar alone in the inhibition of CaM. Chen and colleagues previously reported that erianin could provoke increased Ca^2+^ and Fe^2+^ levels by triggering Ca^2+^/CaM signaling. They established that erianin activated ferroptosis in lung cancer cells via starting Ca^2+^/CaM signaling, and the stoppage of it incredibly reduced cell death provoked by erianin treatment via repressing ferroptosis (30).

Ketoprofen-RGD was more effective than Nar at inhibiting PDE1, essential for signal transduction pathways controlling proliferation, apoptosis, and inflammation (31).

The combination of ketoprofen-RGD and Nar had the highest Ki, followed by ketoprofen-RGD and Nar alone. It was also revealed that the two agents together elevated the Km of PDE1 beyond any effect on the Vmax of the reaction. Ketoprofen-RGD increased Km more than Nar, showing a competitive inhibition mode in blocking PDE1 activity. ΔG values are in line with the above results. Because of its interactions with ketoprofen-RGD and Nar, the reduction in CaM resilience could be associated with decreased G (H_2_O) values. The order of decreased ΔG (H_2_O) values is as follows: Ketoprofen-RGD + Nar > ketoprofen-RGD > Nar. This revealed that ketoprofen-RGD, in combination with Nar, more efficiently reduced CaM stability than the two compounds alone. The concentration of ketoprofen-RGD is lower than Nar regarding CaM, which may be representative of the higher affinity of ketoprofen-RGD for CaM. Our MTT assay results also demonstrated the higher efficiency of the combination of ketoprofen-RGD and Nar in suppressing the K562 and SKOV3 cells’ proliferation than the single agent. Although elevated intracellular levels of cAMP can impede cancer cell proliferation (32), our results affirmed that both ketoprofen-RGD alone and in combination with Nar increased PKA activity and cAMP levels. However, the combination of the two compounds had a more significant effect on these parameters. Our results align with those of Li et al., who demonstrated Nar had a considerable inhibitory influence on the growth of K562 cells (27). However, at lower concentrations, it did not display a cytotoxic influence on normal polymorphonuclear leukocytes (25).

Furthermore, to investigate the potential relationship between PKA activation and the inhibition of K562 and SKOV3 cells by ketoprofen-RGD and Nar, the cells were first treated with Rp-cAMP and then exposed to ketoprofen-RGD + Nar and ketoprofen-RGD alone. The results showed that in the presence of Rp-cAMP, the suppressive impacts of ketoprofen-RGD + Nar and ketoprofen-RGD alone on K562 and SKOV3 cells were reduced compared to the control groups. This indicated that the growth-inhibitory activities of the mentioned drugs were moderated by increasing cAMP levels and the consequent PKA activation. As previously stated, promoting inflammatory processes in cancers is a detriment to cancer progression. Thus, it is very important to modulate these responses in cancer therapy. Therefore, we pretreated K562 and SKOV3 cells with LPS as an inducer of an inflammatory response. The cells were then treated with ketoprofen-RGD plus Nar, ketoprofen-RGD alone, and FSK. The results demonstrated the anti-inflammatory activities of both drug regimens by suppressing TNF-α, IL-1β, IL-6, and IFNγ levels. These data were compared with the results of the FSK treatment. FSK is an adenylate cyclase activator that acts by increasing the levels of intracellular cAMP (33).

Besides, IκBα is a crucial regulator of the transcription factor NF-κB, and deregulation of IκBα cellular levels impacts a variety of diseases, including chronic inflammatory diseases and cancers (34, 35). Western blotting results revealed that ketoprofen-RGD increased IκBα protein expression and counteracted the suppressive effects of LPS. Additionally, Nar potentiated the effect of ketoprofen-RGD on IκBα expression and reduced IκBα degradation.

Cancer progression may occur due to immunosuppression induced by local cytokines, which can lead to the expansion of tumors as a mechanism of escape (36, 37). The present study revealed for the first time that ketoprofen-RGD and Nar seemed to exert their anti-inflammatory impacts on K562 and SKOV3 cells by elevating the concentration of cAMP molecules in these cancer cells.

### 5.1. Conclusions

These findings showed that Nar could improve the antagonizing consequences of ketoprofen-RGD on CaM proteins. These combinations hindered PDE1 and therefore improved the cAMP status and PKA action by antagonizing CaM. The combination of ketoprofen-RGD and Nar and ketoprofen-RGD alone diminished the viability of K562 and SKOV3 cells, maybe through the cAMP/PKA pathway by inhibiting PDE1 and CaM. The present data also confirmed the anti-inflammatory activities of both drugs by modulating the cAMP pathway. Therefore, they can be anti-proliferative representatives, necessitating investigating their probable anticancer consequences in prospective research.
